# Interaction of prion protein with acetylcholinesterase: potential pathobiological implications in prion diseases

**DOI:** 10.1186/s40478-015-0188-0

**Published:** 2015-04-03

**Authors:** Joan Torrent, Alba Vilchez-Acosta, Diego Muñoz-Torrero, Marie Trovaslet, Florian Nachon, Arnaud Chatonnet, Katarina Grznarova, Isabelle Acquatella-Tran Van Ba, Ronan Le Goffic, Laetitia Herzog, Vincent Béringue, Human Rezaei

**Affiliations:** Institut National de la Recherche Agronomique, UR892, Virologie Immunologie Moléculaires, Jouy-en-Josas, F-78350 France; Laboratori de Química Farmacèutica (Unitat Associada al CSIC), Facultat de Farmàcia, and Institut de Biomedicina (IBUB), Universitat de Barcelona, Barcelona, E-08028 Spain; Département de Toxicologie, Institut de Recherche Biomédicale des Armées, Brétigny-sur-Orge, F-91223 France; Institut National de la Recherche Agronomique, UMR866, Université Montpellier 1, Dynamique Musculaire et Métabolisme, Montpellier, F-34060 France; Université Montpellier 2, Montpellier, F-34095 France; Inserm, U710, Montpellier, F-34095 France; EPHE, Paris, F-75007 France

**Keywords:** Amyloid, Protein misfolding, Protein aggregation, Neurodegeneration

## Abstract

**Introduction:**

The prion protein (PrP) binds to various molecular partners, but little is known about their potential impact on the pathogenesis of prion diseases

**Results:**

Here, we show that PrP can interact *in vitro* with acetylcholinesterase (AChE), a key protein of the cholinergic system in neural and non-neural tissues. This heterologous association induced aggregation of monomeric PrP and modified the structural properties of PrP amyloid fibrils. Following its recruitment into PrP fibrils, AChE loses its enzymatic activity and enhances PrP-mediated cytotoxicity. Using several truncated PrP variants and specific tight-binding AChE inhibitors (AChEis), we then demonstrate that the PrP-AChE interaction requires two mutually exclusive sub-sites in PrP N-terminal domain and an aromatic-rich region at the entrance of AChE active center gorge. We show that AChEis that target this site impair PrP-AChE complex formation and also limit the accumulation of pathological prion protein (PrP^Sc^) in prion-infected cell cultures. Furthermore, reduction of AChE levels in prion-infected heterozygous AChE knock-out mice leads to slightly but significantly prolonged incubation time. Finally, we found that AChE levels were altered in prion-infected cells and tissues, suggesting that AChE might be directly associated with abnormal PrP.

**Conclusion:**

Our results indicate that AChE deserves consideration as a new actor in expanding pathologically relevant PrP morphotypes and as a therapeutic target.

**Electronic supplementary material:**

The online version of this article (doi:10.1186/s40478-015-0188-0) contains supplementary material, which is available to authorized users.

## Introduction

The prion protein (PrP) plays a key role in the pathogenesis of transmissible spongiform encephalopathies, also known as prion diseases [1,2]. These fatal and infectious neurodegenerative conditions are caused by prions, which consist mainly, if not exclusively, of misfolded pathological conformers (PrP^Sc^) of the host cellular PrP isoform (PrP^C^) [3].

While the massive contribution of PrP conformational changes to the etiology of prion diseases is widely recognized, less is known about the role of other molecules that interact directly with PrP. The large number of potential PrP^C^-interacting molecules [4-7] suggests that PrP^C^, which is preferentially localized in lipid rafts, is a key member of a multi-protein complex at the plasma membrane. Moreover, results obtained using the protein misfolding cyclic amplification procedure are consistent with an essential role of auxiliary macromolecules in PrP conformational rearrangements, leading to prion infectivity and strain maintenance [8-10]. It is therefore reasonable to hypothesize that in specific cellular environments in the nervous and lymphoreticular systems, interaction with a common molecular partner might promote the selection and enrichment of thermodynamically and conformationally unique PrP morphotypes among the plethora of structurally distinct species predicted by the high-dimensional PrP folding energy landscape [11]. These PrP morphotypes may lie at the origin of prion diseases.

To investigate the influence of a biological factor on PrP structural changes and morphotype selection, we focused on acetylcholinesterase (AChE), a widely distributed protein in mammalian tissues and fluids with a large spectrum of functions. Like PrP^C^, AChE essentially concentrates in the lipid raft fraction of the plasma membrane and is found in two different tissues where prion can be replicated: the synaptic terminals in cholinergic and non-cholinergic brain areas and reticuloendothelial cells of lymphoid organs [12,13]. There is compelling evidence that beyond the well-known hydrolysis of the neurotransmitter acetylcholine in cholinergic synapses, AChE exerts a variety of less-characterized “non-classical” roles (attributed to a non-enzymatic activity or to its cholinergic action in non-neural tissues) in stress responses and immunity, neuritogenesis and neurodegeneration [14-17]. Indeed, AChE might contribute to Alzheimer’s disease (AD) pathogenesis through its ability to interact with amyloid β (Aβ) and to increase neuronal death [18-20], the amyloid burden and cognitive deficits in mice [21,22]. Aβ pro-aggregating activity appears to be mediated by the peripheral anionic site (PAS) of AChE [23], located at the mouth of a 20 Å deep narrow gorge, at the bottom of which is placed the catalytic site [24,25]. Hence, AChE inhibitors (AChEis), designed to interact with both the active and PAS sites of AChE, strongly inhibit not only AChE catalytic activity, but also AChE-induced Aβ assembly [26-29]. Recent evidence indicates that AChE also mediates the assembly of two synthetic peptide fragments spanning residues 106–126 and 82–146 of PrP and that these interactions are impaired by AChEis that specifically bind to the PAS site [30,31]. Nevertheless, the influence of AChE in full-length PrP conformational changes, and its functional link to prion replication and pathogenesis remain to be determined. Here we addressed the hypothesis that a broader non-enzymatic function of AChE in PrP misfolding and aggregation could be relevant in prion diseases. Indeed, reduced AChE activity in lumbar CSF samples and altered AChE glycosylation in the brain and CSF samples of patients with Creutzfeldt-Jakob disease (CJD) have been observed [32,33]. Therefore, we investigated the role of AChE in prion pathogenesis, focusing both on its effect on PrP aggregation and PrP-mediated toxicity. First we found that two established AChEis hindered PrP^Sc^ accumulation in cell cultures. Using biophysical and biochemical approaches, we then demonstrate that AChE binds to both monomeric and amyloid fibril conformers of full-length recombinant PrP. We show that the resulting hetero-assemblies have higher cytotoxicity. Once recruited into PrP fibrils, AChE loses its enzymatic activity. The use of different truncated PrP variants revealed that AChE directly interacts with PrP N-terminal domain. Through AChEi reduction of AChE binding to both PrP conformers, we demonstrate that an aromatic-rich hotspot in the AChE active center gorge entrance is involved in the formation of the heterologous complex. Moreover, reduced AChE levels in prion-infected heterozygous AChE knockout (AChE^+/−^) mice led to significantly longer survival compared to wild type controls. Finally, we found that AChE levels were altered in brain and spleen of prion-infected mice and also in infected cell cultures compared to non-infected controls.

## Materials and methods

### Recombinant PrP and AChE

Full-length human PrP and different His-tagged truncated forms (∆23-99, ∆100-120, ∆23-99, ∆121-231 and Δ23-120) were produced in *Escherichia coli* and purified as described previously [34]. Purified monomeric PrPs were stored lyophilized and recovered in the desired buffer by elution through a G25 desalting column (GE Healthcare). Full-length human AChE was expressed in Chinese hamster ovary (CHO) cells and purified from cell culture medium as described previously [35]. Purified dimeric AChE was concentrated using a centricon-30 ultrafiltration micro-concentrator from Amicon (Millipore Corporation, Billerica, MA, USA) and stored at 4°C. All concentrations given for fibrillar PrP and dimeric AChE refer to the respective equivalent monomer concentration.

### AChEis

Racemic huprine Y and Hup8TH were prepared in the form of hydrochloride salts as previously described [36,37], whereas tetrahydroaminoacridine hydrochloride (tacrine), huperzine A and propidium iodide were purchased from Sigma-Aldrich.

### Cell culture

MovS6 cells are immortalized neuroglial cells isolated from transgenic mice that express ovine PrP [38]. Cells were grown in Opti-MEM medium with L-glutamine supplemented with 10% fetal calf serum, 1% penicillin–streptomycin. Cell cultures were infected at 80% confluence in 12-well plates with the 127S strain of sheep scrapie (50 ml of 0.2% (w/v) brain homogenate of terminally ill *tg338* mice in 2 ml of culture medium) as described in [39]. Four days after exposure, cells were carefully rinsed and passaged at a 1:10 dilution in 25-cm^2^ flasks (passage 1). Cells were further incubated and diluted 1:10 at each following passage.

### PrP^Sc^ clearance assay and immunoblotting

Infected MovS6 cells (~10^6^/25 cm^2^ flasks) were incubated with various AChEis at different final concentrations for 6 days. At confluence, cells were lysed and treated as described in [38]. Cell viability was assayed using the (3-[4,5-dimethylthiazol-2-yl]-2,5- diphenyltetrazolium bromide; thiazolyl blue) MTT reduction assay (Sigma-Aldrich) according to the manufacturer’s instructions. Western blotting was performed according to standard procedures. The SAF32 monoclonal antibody [40], an IgG against the octarepeat domain, was used to detect PrP^C^; the Sha31 monoclonal antibody (epitope 148–159) [40] was used to detect PrP^res^ on immunoblots. Detection of AChE was done as described above using a rabbit anti-AChE antibody [41]. To confirm equal protein loading, membranes were also probed with the anti-b-actin antibody clone AC-74 (Sigma-Aldrich). Band intensity for PrP^Sc^ was measured using the GeneTools software after acquisition of chemiluminescent signals with a GeneGnome digital imager (Syngene).

### Formation of amyloid fibrils

PrP amyloid fibrils were formed using the manual setup protocol described previously by [42]. Fibril formation was monitored using a ThT binding assay [42]. Samples were dialyzed in 10 mM sodium acetate, pH 5.0. Then fibrils were collected by ultracentrifugation and resuspended in 10 mM sodium acetate, pH 5.0. A washing step was performed by repeating the ultracentrifugation and resuspension steps.

### Transmission Electron Microscopy (TEM)

Samples were deposited on Formvar carbon-coated grids, negatively stained with freshly filtered 2% uranyl acetate, dried and viewed using a JEOL 1200EX2 electron microscope (JEOL USA, Inc, Peabody, USA). For immunogold labeling, samples adsorbed onto grids and air-dried were washed with H_2_O. Non-specific binding was blocked by incubation in PBS with 1% (w/v) bovine serum albumin (BSA) for 15 min. Grids were then placed onto a droplet of H-134 anti-AChE polyclonal antibody (Santa Cruz Biotechnology, Inc. Heidelberg, Germany) diluted 1/25 in PBS with 1% (w/v) BSA for 1 h. Grids were then washed in three droplets of PBS with 1% (w/v) BSA (4 min/each) and placed on a droplet of anti-rabbit IgG conjugated to 10 nm colloidal gold particles for 45 min (Sigma-Aldrich) diluted 1/50 in PBS with 0.1% (w/v) BSA. Before viewing, grids were washed with H_2_O and negatively stained with freshly filtered 2% uranyl acetate.

### Fluorescence measurements

Fluorescence measurements were performed at 20°C using a FP-6200 fluorimeter (Jasco France, Bouguenais, France). Aliquots of each protein sample were incubated with 10 μM thioflavin T (ThT) for 1 min or 50 μM 8-anilino-1-naphthalene sulfonate (ANS) at room temperature for 10 min before fluorescence measurements. For ANS spectra, excitation was at 385 nm. ThT emission spectra were recorded after excitation at 450 nm. The excitation and emission slit widths were 4 nm.

### Static light scattering (SLS)

Static light scattering kinetic experiments were performed with a homemade device using a 407-nm laser beam. Light-scattered signals were recorded at a 112° angle. Signals were processed with a homemade MatLab program. This technique was coupled with size-exclusion chromatography, to estimate the absolute molecular weight of AChE (oligomeric state) using the relationship between the intensity of light scattered by the molecule and its molecular weight and concentration, as described by the Rayleigh theory. A 60 × 0.78 cm TSK4000SW column (Tosoh Bioscience, Worcestershire, UK) was used with 20 mM Mes pH 6.0 as elution buffer. The temperature of the column was maintained at 18°C. Carbonic anhydrase was used as standard protein for calibration purposes.

### Differential scanning calorimetry (DSC)

Differential scanning calorimetry experiments were performed using a VP-DSC MicroCalorimeter device (GE Healthcare) with a temperature scan rate of 1°C/min. Thermograms were recorded using 50 μM PrP in the presence or not of 50 μM Hup8TH. To ensure reversible thermal denaturation conditions, 10 mM sodium citrate pH 4.6 was used.

### BS^3^ cross-linking experiments

Cross-linking was induced at 25°C by the addition of BS^3^ (500 μM final concentration) (Thermo Fisher Scientific, Rockford, IL, USA) in a solution of 10 μM PrP, 5 μM AChE or a mix of both proteins in 20 mM Hepes, pH 7.0. Reactions were stopped after 10 min by adding an excess of amine groups with 50 mM Tris–HCl buffer. After addition of Laemmli buffer, samples were heated at 100°C for 10 min and separated by SDS-PAGE on 12% Criterion™ XT Bis-Tris Precast Gels (Bio-Rad) followed by silver staining.

### Fibril annealing and PK digestion assay

PrP fibrils in the presence or absence of AChE were treated as described previously [43]. Samples were heated in denaturing sample buffer (60 mM Tris–HCl, 2% SDS, 5% β-mercaptoethanol, 2.25 M urea) at 95°C for 15 min and separated on 12% Criterion™ XT Bis-Tris Precast Gels (Bio-Rad) followed by silver staining. Quantification of protein-band intensities was performed by densitometric analysis using NIH ImageJ software (National Institutes of Health, Bethesda, MD).

### Primary neuronal cells

Primary cell cultures of neurons were derived from the cerebral cortex of 17.5 E mouse embryos and carried out as reported previously [44]. 20 μM of recombinant PrP isoforms (soluble, fibrils) were pre-incubated in 10 μM AChE at 37°C for 30 minutes before their addition to the cells at a final concentration of 1 and 0.5 μM, respectively. After 72 hours *in vitro*, the cytotoxic effect of each treatment was analyzed by fixing cells in 4% paraformaldehyde solution and staining with Hoechst 33258. Apoptotic cells were identified by the characteristic nuclear bright blue fluorescence due to condensed or fragmented chromatin. Neurons were identified with a primary mouse anti-beta 3 tubulin antibody (1:400; Sigma-Aldrich) and revealed by staining with an anti-mouse Cy3 secondary antibody (1:400; Jackson ImmunoResearch Europe Ltd., Suffolk, UK). Digital images were captured from 10 random fields for each sample (≈3500 cells total) using an Axiovert 200 M Zeiss inverted microscope (Carl Zeiss S.A.S., Marly le Roi, France). Neuronal cell death was determined by counting the bright neurons and expressed as the percentage of the total cell number compared with control cultures. Data were analyzed by one-way analysis of variance and a Tukey test was carried out on the results of four independent experiments.

### Inhibition of AChE enzymatic activity

7.3 nM AChE was mixed with PrP fibrils (concentration ranging from 8 to 25 μM) or 25 μM monomeric PrP at 37°C in 100 mM sodium phosphate buffer pH 7.4 containing 0.33 mg/mL BSA. The residual activity contained in a 10-μL aliquot was monitored at 25°C and pH 7.4, according to the Ellman’s method, using 1 mM acetylthiocholine and 0.5 mM dithiobisnitrobenzoic acid and a final volume of 200 μl.

### Ethics statement

Animal experiments were carried out in strict accordance with the EU directive 2010/63 and were approved by the authors’ institution local ethics committee (Comethea; permit number 12/034).

### Infection of *tg338* mice with LA21K *fast* prions

The cloned ovine prion strain LA21K *fast* used in this study has been previously described [45]. Individually identified 6 to 10 week/old *tg338* mice were inoculated intracerebrally with 20 μl of 10% (w/v) infected brain homogenates (prepared in 5% glucose) (n = 5 mice). Age-matched control *tg338* mice received uninfected brain homogenates (n = 5 mice). Mice showing prion-specific neurological signs were monitored daily and euthanized at the terminal stage of prion disease. At this time point, control mice were also euthanized. After perfusion with physiological saline and euthanasia, half-brain specimens were dissected into two parts: a cortex-enriched fraction and a fraction containing the remaining brain areas. These samples were flash frozen, and stored at −80°C. Spleens were also rapidly removed, flash frozen, and stored at −80°C.

### Infection of AChE^*+/−*^ and AChE^+/+^ mice with 139A prions

AChE^+/−^ mice (Xie et al. 2000) and wild type (AChE^+/+^) littermates were maintained by breeding heterozygous males and females (129/Sv genetic background). Individually identified, 6 week/old mice were infected by intracerebral inoculation with 20 μl of a 0.1% (w/v) 139A mouse scrapie strain, as described above.

### Tissue homogenization

Brain and spleen tissues (10% (w/v) and 5% (w/v), respectively) were homogenized using a Rybolyser Precellys (Bertin Technologies, Montigny-le-Bretonneux, France) and ice-cold extraction buffer (25 mM Tris–HCl, 150 mM NaCl, pH 7.3) containing 1% Triton X-100 and protease inhibitors (10 mM EDTA, 40 μg/ml leupeptin, 10 μg/ml pepstatin and 2 mM benzamidine). Extracts were incubated on ice for 1 h and then centrifuged at 30,000 × *g* for 30 min. The protein content of supernatants was determined using the Bicinchoninic acid assay reagent kit (Pierce Biotechnology, Rockford, USA).

### Detection of PrP^Sc^ in brain and spleen

Before the centrifugation step of the homogenization procedure, PrP^**Sc**^ was extracted with the BioRad TsSeE purification and detection kit (Marnes-la-Coquette, France) and digested with 200 μg/ml proteinase K (Euromedex, Mundolsheim, France) at 37°C for 10 min. After denaturation in Laemmli buffer at 100°C for 5 min, samples were run on 12% Criterion™ XT Bis-Tris Precast Gels (Bio-Rad), electrotransferred to nitrocellulose membranes, and probed with the biotinylated anti-PrP monoclonal antibody Sha31, as previously described [46]. Immunoreactivity was visualized by chemiluminescence (GE Healthcare, Orsay, France).

### AChE detection in brain and spleen

200 mg of total proteins were separated on 7.5% Criterion™ TGX™ Gels (Bio-Rad) in reducing conditions, and then transferred to nitrocellulose membranes. Blots were blocked in Tris-buffered saline (TBS) with 0.5% Tween 20 (TBS-T) containing 5% nonfat dried milk at 4°C overnight before incubation with a rabbit anti-AChE antibody [41], (1:2000 in the same TBS-T buffer) at 4°C overnight. Blots were then incubated with peroxidase-conjugated anti-rabbit IgG antibodies (1:10,000; Abliance SAS, Compiègne, France) at room temperature for 30 min, washed extensively and exposed to the chemiluminescent substrate ECL Plus (GE Healthcare). The amount of AChE present in each tissue was determined with the GeneTools software after acquisition of the chemiluminescent signals with a GeneGnome digital imager (Syngene, Frederick, Maryland, USA). To confirm equal protein loading, membranes were also probed with an anti-β-actin (clone AC-74; Sigma-Aldrich, Saint-Quentin Fallavier, France), or anti-GAPDH antibody (Chemicon International, Temecula, USA). AChE protein values were normalized by the corresponding β-actin and GAPDH protein content.

### Determination of AChE activity in brain and spleen

Total AChE activity in the homogenates was assayed using 1 mM acetylthiocholine and 0.5 mM 5,5-dithiobis(2-nitrobenzoic acid) (DTNB) in the presence of 50 μm tetra(monoisopropyl)pyrophosphortetramide (iso-OMPA) (Sigma-Aldrich). Extracts were first incubated in the absence of acetylthiocholine for at least 20 min to block butyrylcholinesterase and saturate the free sulfhydryl groups that interact with DTNB. Changes in optical density were measured at 414 nm.

### RNA extraction and RT-qPCR

Total RNAs were extracted from spleen and brain samples after homogenization in RLT buffer (RNeasy kit, Qiagen) and QIAzol Lysis Reagent (Qiagen), respectively, following the manufacturer’s recommendations. Reverse-transcriptions were performed using the Superscript reverse transcriptase (Invitrogen) and random hexamers (Fermentas). The levels of choline acetyltransferase (ChAT) transcripts and of alternatively spliced AChE transcripts were measured using the Mastercycler realplex sequence detector (Eppendorf) and the double stranded DNA-specific dye SYBR Green system (Applied Biosystems) with the following primers: AChE (exon2) sense 5′-GCATACACCTTCCCTGGCTT-3′, antisense 5′-AAAAGCTGAGACTGGGCCTC-3′; AChE-E sense 5′-TCAGCGCCACCGCCACGGAG-3′, antisense 5′-AGAGGAGGGACAGGGCTAAG-3′; AChE-S sense 5′-TGTGAGCCTGAACCTGAAGC-3′, antisense 5′-CTGGTTCTTCCAGTGCACCA-3′; AChE-R sense 5′-TCAGCGCCACCGGTAGGCGC, antisense 5′-AGAGGAGGGACAGGGCTAAG-3′; ChAT sense 5′-AAATGGCGTCCAACGAGGAT-3′, antisense 5′-GCTCGATCATGTCCAGGGAG-3′; HPRT sense 5′-GGTTAAGCAGTACAGCCCCA-3′, antisense 5′-TCCAACACTTCGAGAGGTCC-3′. PCR conditions and cycles were as follows: initial DNA denaturation at 95°C for 10 min, followed by 40 cycles at 95°C for 15 sec, then an annealing step at 60°C for 15 sec, and finally an extension step at 72°C for 30 sec. To ensure that the primers produced a single and specific PCR amplification product, a dissociation curve was performed at the end of each PCR assay. Relative quantification was performed by using the comparative ΔΔCt method. The mean ΔCt obtained using samples from mock-infected mice for each isoform was used as calibrator after normalization to the endogenous control hypoxanthine-guanine phosphoribosyltransferase (HPRT). Results are presented as n-fold difference relative to the calibrator (RQ = 2^-ΔΔCt^).

## Results

### The potent tight-binding AChEis huprine Y and Hup8TH limit PrP^Sc^ accumulation in prion-infected cell cultures

First we asked whether AChE actively participates in PrP^Sc^ accumulation. We thus investigated the effect of AChE inhibition in prion-infected MovS6 cells [38], a model previously used in proof of principle studies on candidate anti-prion agents [47,48]. We chose the high-affinity reversible AChEis huprine Y (a huperzine A-tacrine hybrid) [36,49] and Hup8TH (a huprine-tacrine heterodimer) [37], based on their extremely potent inhibition of AChE catalytic activity and particularly their ability to impair AChE-induced aggregation of Aβ and PrP peptides [30,31]. In parallel, we also tested the parent compounds tacrine and huperzine A, as well as propidium iodide, a specific AChE PAS inhibitor.

**Figure 1:**
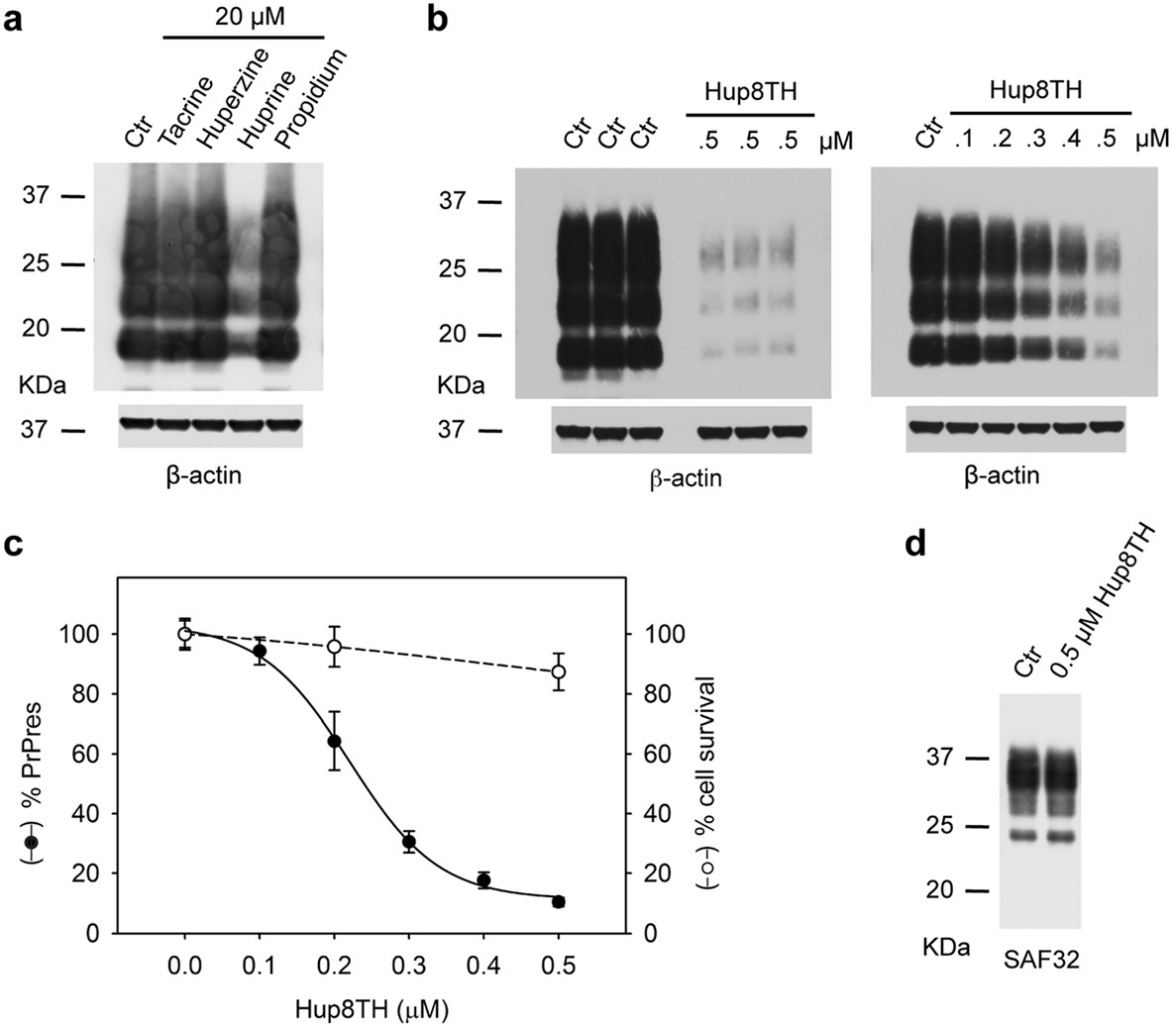
**The tight-binding AChEis huprine Y and Hup8TH efficiently decrease PrP** ^**Sc**^ **accumulation in Scrapie-infected MovS6 cells. a** Scrapie-infected MovS6 cells were incubated with 20 μM of the indicated AChEis for 6 days and then lysed. PrP^Sc^ accumulation was determined by using the anti-PrP antibody Sha31b; the anti-β-actin antibody was used to control protein loading. Ctr, vehicle-treated cells. **b** Prion-infected MovS6 cells were incubated with 0.5 μM Hup8TH or 0.1 to 0.5 μM Hup8TH and then processed as in a. **c** Densitometric measurement of PrP^Sc^ signal, expressed as percentage relative to the untreated control (closed circles). Results are expressed as the mean ± SD of three different experiments. Cell viability determined by using the MTT assay is also shown (open circles). The absorbance values are expressed as the mean ± SD of four independent experiments relative to the absorbance values of the untreated control. Solid and dashed lines are fits to the data. **d** Protein lysates from uninfected MovS6 cells were analyzed by immunoblotting without proteinase K digestion. The effect of Hup8TH on PrP^C^ accumulation was determined using the SAF32 antibody. Molecular weights (in kilodaltons) are indicated on the left of the blots.

Western blot analysis after PK digestion of crude extracts from confluent, prion-infected MovS6 cells cultured in the presence of 20 μM of each AChEi or vehicle alone for 6 days showed that only huprine Y inhibited PrP^Sc^ accumulation (Figure 1a). As Hup8TH was cytotoxic at this concentration, we repeated the experiment with non-cytotoxic doses (0.1 to 0.5 μM). Hup8TH treatment reproducibly led to PrP^Sc^ level reduction and this effect was dose-dependent (Figure 1b). These results reveal that AChEis limit PrP^Sc^ accumulation in an AChE-binding depending manner, i.e. tacrine (no effect) < huprine Y < Hup8TH. For Hup8TH, fitting of the dose–response curve (Figure 1c) yielded a 50% inhibitory concentration (IC_50_ value) of approximately 0.22 ± 0.01 μM. The toxicity of Hup8TH was assessed using the MTT metabolic assay (Figure 1c). Cell viability decreased by approximately 10% at concentrations of Hup8TH required for maximal inhibition of PrP^Sc^ accumulation. In uninfected MovS6 cells, PrP^C^ expression levels were unaffected by 0.5 μM Hup8TH (Figure 1d), ruling out the possibility that Hup8TH inhibition of PrP^Sc^ accumulation in infected culture was due to an altered steady state level of PrP^C^.

### AChE binds to PrP monomers and induces their aggregation

**Figure 2:**
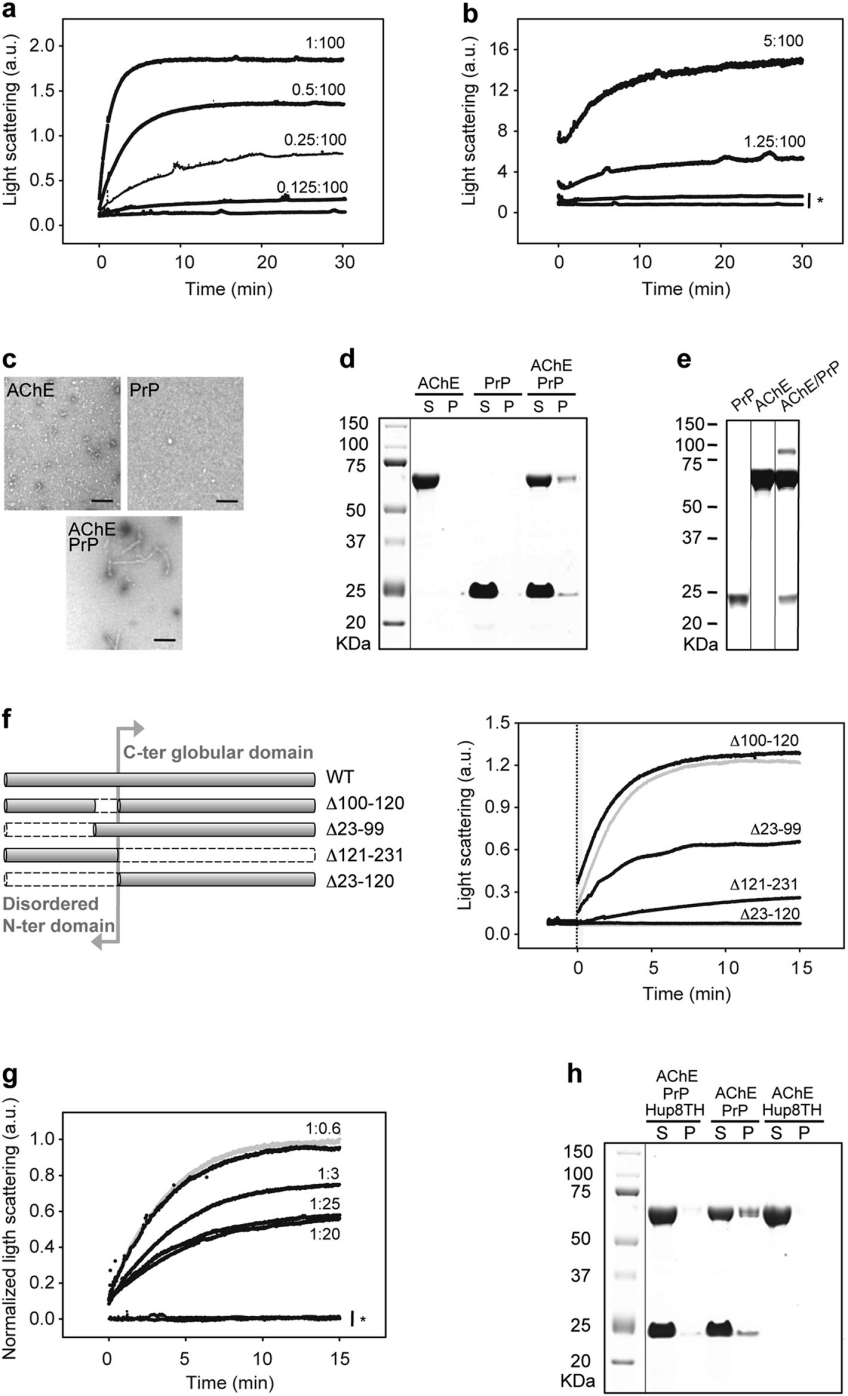
**The formation of an aggregation-prone complex via specific interaction of AChE with PrP N-terminal domain is partially inhibited by Hup8TH. a** Kinetics of AChE-PrP assembly. Increasing concentrations of AChE were added to 5 μM PrP. AChE:PrP molar ratios are indicated next to the trace. The extent of protein aggregation was recorded by light scattering intensity (also in panels **b**, **f** and **g**). The results for AChE in the absence of PrP are not shown because they overlap with the kinetic profile of PrP alone. **b** Probing the specificity of the interaction by strengthening the ionic character of the buffer. AChE was added to 20 μM PrP in the presence of 200 mM NaCl. The AChE:PrP molar ratios are indicated next to the trace. The asterisk shows the kinetic profiles of AChE (lower curve) and PrP (higher curve) alone. **c** Negative-stained transmission electron micrographs of AChE (upper left panel), PrP (upper right panel) and a mix of both proteins (lower panel). The molar ratio used was 0.5:1 (AChE:PrP). Scale bar = 250 nm. **d** The presence of both PrP and AChE in the insoluble fraction (pellet, P) after centrifugation of a mixture of 10 μM PrP with 5 μM AChE (incubated at 37°C for 30 min) confirms the formation of PrP-AChE aggregates. Gels were stained with Coomassie Blue. S, soluble fraction. **e** BS^3^ cross-linking assay demonstrates the formation of a heterodimer. Silver-stained gel showing PrP (10 μM), AChE (5 μM), and a mix of both proteins after incubation with 500 μM BS^3^. **f** Importance of the PrP N-terminus for the heterologous interaction. Left panel: schematic representation of the various PrP constructs. Right panel: kinetics of AChE assembly with truncated PrP variants. 50 nM AChE was added to 2 μM PrP variants (the full-length PrP profile is shown as grey line). The vertical dotted line indicates when AChE was added (time zero). **g** Hup8TH decreases AChE binding to PrP. AChE was pre-incubated at room temperature with increasing concentrations of Hup8TH for 30 min (AChE:Hup8TH molar ratios from 1:0.6 to 1:25). Then, 250 nM PrP was incubated with 50 nM pre-treated or not (grey line) AChE (AChE:Hup8TH molar ratios are indicated next to the trace). The asterisk highlights the kinetic profiles of AChE and PrP alone incubated with the highest Hup8TH concentration. **h** The effect of Hup8TH on AChE-PrP co-aggregation was monitored by Coomassie blue staining after SDS-PAGE. 10 μM PrP and 5 μM AChE were incubated or not at 37°C with Hup8TH (1:100 molar ratio, AChE:Hup8TH) for 30 min. Supernatant and pelleted fractions obtained after centrifugation were analyzed**.** Experiments were performed in 20 mM Mes buffer (pH 6.0) at 37°C; a.u., arbitrary units. Molecular weights (in kilodaltons) are indicated to the left of the blots.

To determine whether a direct AChE/PrP interaction could explain these results, we performed SLS measurements of full length PrP mixed with increasing AChE concentrations (Figure 2a). The raise in scattering intensity upon mixing the two proteins (time zero) indicated an increase in the average molecular weight of the sample, suggesting the formation of AChE:PrP complexes. After strengthening the ionic character of the buffer by adding 200 mM NaCl, the reaction was readily detected even at the molar ratio of 1.25:100 (AChE:PrP), confirming the interaction specificity (Figure 2b). TEM also supported AChE positive effect on PrP aggregation by showing that larger PrP assemblies were formed in the presence of AChE than in its absence (Figure 2c).

To assess whether the aggregates (identified by SLS) resulting from the interaction between PrP and AChE contained both proteins, we fractionated the solutions into supernatant (S) and pellet (P) fractions by ultracentrifugation and separated them by SDS-PAGE (Figure 2d). PrP and AChE alone were completely soluble, whereas, after mixing, 10-20% of the total content was present in the pellet.

To further substantiate AChE-PrP interaction, we then performed chemical crosslinking using the amine-to-amine cross-linker BS^3^ followed by SDS-PAGE (Figure 2e). After crosslinking, PrP and AChE alone showed a unique band that corresponded to the monomeric protein. Conversely, in the mix, an additional band was visible and its molecular weight was consistent with the assembly of one PrP monomer with one AChE monomer.

To characterize the specific regions within the PrP molecule that are crucial for AChE binding, we generated various PrP truncated variants (Figure 2f). In the absence of the largely unstructured N-terminal region of PrP (residues 23–120), AChE-mediated aggregate formation was abrogated. All other PrP variants allowed aggregation, albeit with varying efficacy. These results led us to conclude that AChE directly interacts with PrP unstructured N-terminus and more precisely with two mutually exclusive sub-sites that are included between residues 23 and 99 and residues 100 and 120.

### Hup8TH reduces AChE-induced aggregation of PrP

We then assessed whether Hup8TH, the most potent anti-PrP^Sc^ AChEi in cell culture, could interfere with AChE-PrP interaction, and consequently reveal the existence of a hot spot in AChE for the binding interface. To this aim, we pre-incubated AChE with Hup8TH at molar ratios up to 1:25 (AChE:Hup8TH) at room temperature for 30 min and then added the solution to monomeric PrP (time zero). SLS measurements (Figure 2g) indicated that Hup8TH inhibited the aggregation by approximately 50% at the highest dose tested. The compound had no effect on the monitored light scattering of PrP and AChE alone. In addition, to test whether the inhibitory activity of Hup8TH relied on an unexpected direct interaction with PrP, we measured the heat-induced PrP equilibrium unfolding by DSC. To this purpose, we used PrP at pH 4.6, which allows studying the reaction as a reversible two-step process [50]. DSC thermograms of PrP in the absence and in the presence of Hup8TH at equimolar concentration showed no difference in the single endothermic peak typical of protein denaturation (Additional file 1), indicating that Hup8TH does not directly interact with PrP.

To further characterize the inhibitory effect of Hup8TH on AChE-PrP aggregation, we separated samples into pellet and supernatant fractions by ultracentrifugation. SDS-PAGE analysis (Figure 2h) showed that pre-treatment of AChE with Hup8TH practically suppressed the aggregation effect. Moreover, binding of Hup8TH to AChE did not alter the enzyme solubility, excluding any decrease in the soluble AChE pool available for interaction with PrP.

These results validate the specificity of the AChE-PrP hetero-assembly and provide strong evidence for the existence of a hotspot on AChE for PrP binding that can be targeted successfully by a small inhibitor molecule.

### AChE associates with PrP fibrils

**Figure 3:**
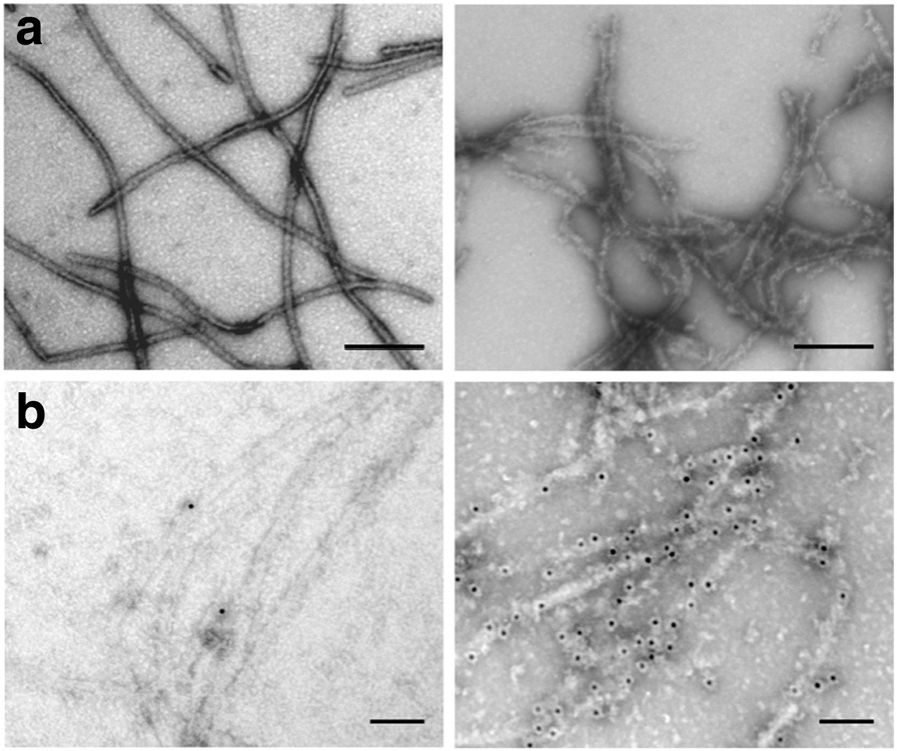
**Macrostructure of the AChE-PrP fibril morphotype.** Negative-stained transmission electron micrographs of PrP fibrils formed in the absence (left panels) or presence (right panels) of AChE, after direct deposition onto a grid **(a)**, or following incubation with an anti-AChE antibody and a secondary antibody conjugated to 10-nm gold nanoparticles **(b)**. The molar ratio used was 0.5:1 (AChE:PrP). All scale bars are 100 nm.

We then studied whether PrP fibril structure could be modulated by AChE. The enzyme did not affect the fibril-formation process promoted by partially-denaturing solvent conditions (2 M guanidine-HCl). Nevertheless, fibrils formed from a mix of AChE and PrP (0.5:1 ratio) (Figure 3a, right panel) were decorated by punctuate material, probably the co-assembled AChE, when compared to fibrils obtained from PrP alone (Figure 3a, left panel).

To confirm the presence of AChE in PrP fibrils, we used immuno-electron microscopy and an anti-AChE antibody that was revealed with a secondary antibody conjugated to 10-nm gold nanoparticles (Figure 3b). Only in fibrils formed in the presence of AChE, electron-dense gold nanoparticles colocalized with PrP fibrils (Figure 3b right panel), demonstrating the interaction of AChE with the fibrils. The AChE-PrP assembly obtained in mild denaturing conditions highlights the specificity of the reaction.

**Figure 4:**
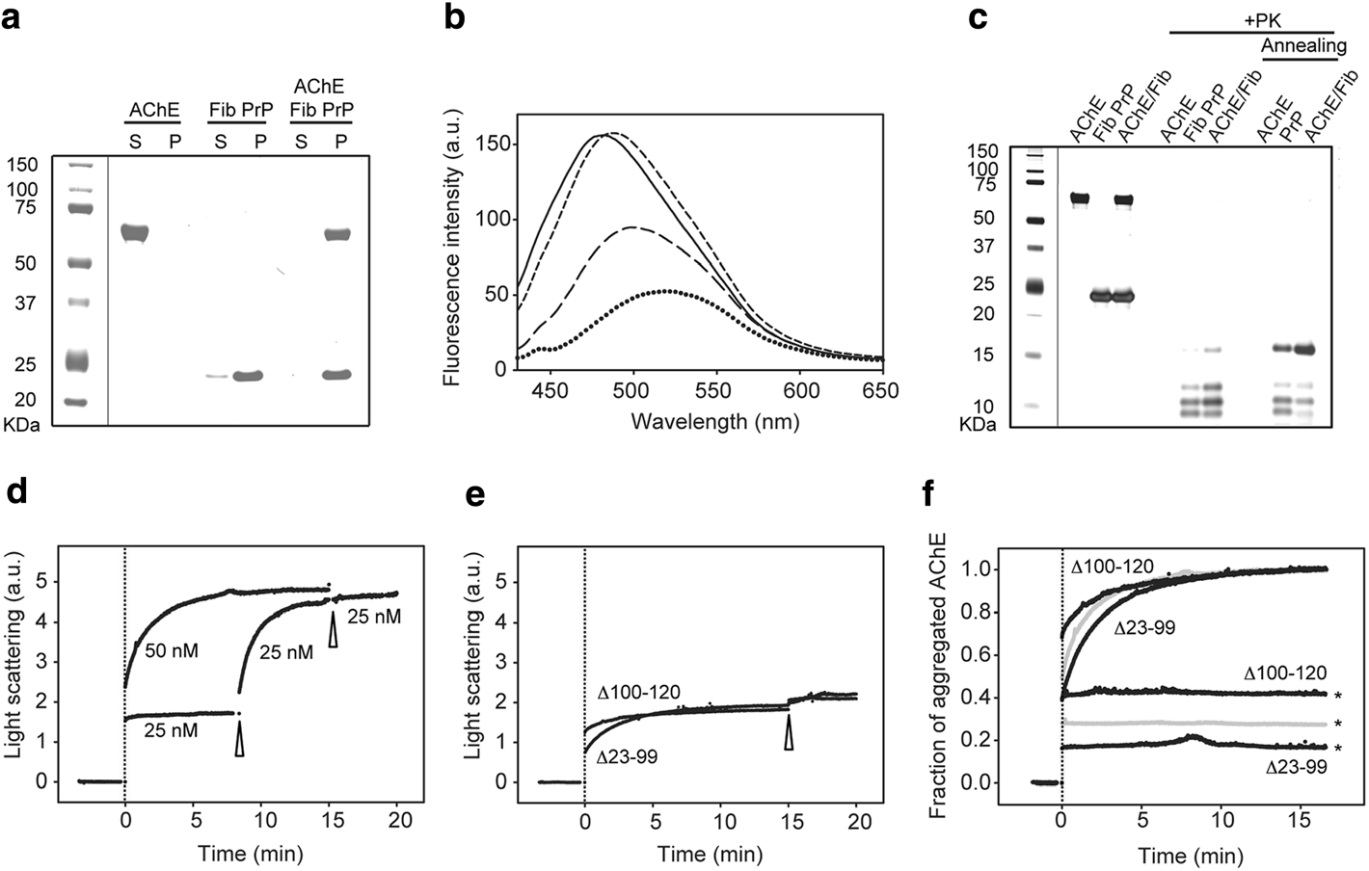
**AChE associates with amyloid fibrils via the PrP N-terminus and affect their physical-chemical characteristics.** This association is inhibited by Hup8TH. **a** Co-aggregation of AChE with preformed PrP fibrils (Fib PrP) was followed by Coomassie blue staining after SDS-PAGE of 10 μM Fib PrP mixed with 5 μM AChE at 37°C for 30 min. **b** AChE increases ANS binding to preformed PrP fibrils. ANS fluorescence emission spectra of monomeric PrP protein (dotted line), untreated PrP fibrils (long dashed line), fibrils after AChE association (short dashed line) and AChE alone (solid line). Samples correspond to either supernatant or pelleted fractions obtained after centrifugation. Final protein concentrations: 10 μM PrP, 5 μM AChE. Incubation conditions: 37°C for 30 min. **c** Altered digestion profile of the PK-resistant core of PrP fibrils after AChE binding. Silver staining following electrophoretic separation of PrP fibrils (10.5 μM ) incubated or not with AChE (0.5 μM), before and after PK digestion (+PK). The annealing procedure involves brief heating of the sample at 80°C in the presence of detergent. **d** Kinetics of AChE-PrP fibril co-assembly. Light scattering intensity was used to follow the increase in the average molecular weight during successive additions (indicated by an arrowhead) of 25 nM AChE to 50 nM preformed PrP fibrils. The vertical dotted line indicates when AChE was first added (time zero). Saturation occurs at equimolar concentrations. The profile obtained after a single addition of 50 nM AChE to the fibrillar sample is also shown to emphasize the equivalent total amplitude change. **e** AChE interacts with PrP fibrils obtained from two N-terminal truncated PrP variants (∆100-120 and ∆23-99) and reaches saturation at half the molar ratio (0.5:1, AChE:PrP), compared to full-length PrP fibrils. Two successive additions (indicated by an arrowhead) of 25 nM AChE to the two truncated PrP fibrils (50 nM) were performed. **f** Hup8TH decreases the binding of AChE to PrP fibrils. AChE was pre-incubated at room temperature with Hup8TH at a molar ratio of 1:20 (AChE:Hup8TH) for 30 min. Then, 50 nM PrP fibrils obtained from full-length PrP were incubated with 50 nM pre-treated (grey line with asterisk) or non-inhibited AChE (grey line without asterisks). The same experiment was performed using the two truncated PrP fibrils and with 25 nM (according to the saturation values observed in e) of pre-inhibited (black lines with asterisks) or not (black lines without asterisks) AChE. Data were normalized between 0 and 1 to easily compare the effects of the AChEi on the fraction of aggregated AChE. Experiments were performed in 20 mM Mes buffer (pH 6.0) at 37°C; a.u., arbitrary units. Molecular weights (in kilodaltons) are indicated to the left of the blots.

Next, to assess whether AChE could bind also to mature pre-formed PrP fibrils, we incubated AChE with preformed PrP fibrils at 37°C for 30 min. After centrifugation, AChE co-sedimented with PrP fibrils (Figure 4a). Analysis by TEM of the macrostructure of this AChE:PrP fibril complex revealed thicker fibrils, compared to PrP fibrils alone, suggesting that AChE homogenously coats the filaments (Additional file 2).

We then evaluated whether AChE binding affected some generic physical properties of PrP amyloid fibrils. First, we used the fluorescence probe ANS to study the presence of hydrophobic clusters on the PrP fibrillar surface (Figure 4b). ANS binding to PrP fibrils resulted in higher fluorescence yield and a blue shift in the fluorescence spectrum, as expected for PrP fibril structures, compared to the weak fluorescence yield observed after ANS binding to soluble PrP [51]. Upon ANS binding to the complex formed between pre-formed PrP fibrils and AChE, the fluorescence spectrum was further enhanced and blue-shifted, showing the presence of additional structured hydrophobic domains. This spectrum was therefore consistent with the observed AChE coating of PrP fibrils.

In addition, we carried out PK digestion to assess alterations in the PrP PK-resistant core after AChE binding (Figure 4c). Compared to PrP amyloid fibrils, the low molecular weight bands in AChE:PrP fibril complexes were more intense (for a densitometric analysis of the corresponding bands see Additional file 3), with a substantial increase of the PK-resistant core and a stronger ~16-kDa PK-resistant band. These differences were further substantiated after annealing, a procedure specific for amyloid PrP structures that induces conformational rearrangements within PrP fibrils [43]. In the AChE:PrP fibril complex, the characteristic 16-kDa band was much intensified, when compared to PrP fibrils alone. These results are consistent with the gain of structural and conformational integrity of PrP fibrils after AChE interaction.

To better understand the mechanism of AChE binding to the PrP amyloid form, we then performed SLS kinetic measurements by successively adding 25 nm AChE to 50 nM of pre-formed PrP fibrils (Figure 4d). An increase in light scattering that reflected the raise in the average molecular weight of the sample was observed, suggesting the formation of a stable complex between AChE and fibrillar PrP. Moreover, the signal was saturated when the molar ratio of 1:1 (PrP:AChE) was reached. As expected, a single addition of an equimolar concentration of AChE (50 nM) had the same effect.

To determine the PrP region involved in the AChE-PrP fibril interaction, we used SLS to investigate the interaction between AChE and two fibrillar samples obtained from two N-terminally truncated PrP variants lacking either residues 23–99 or 100–120, the two mutually exclusive sub-sites for the AChE-PrP interaction that we previously identified using monomeric PrP. AChE bound to both truncated fibrils. Nevertheless, and in contrast to full-length PrP fibrils that contain both sub-sites, the kinetic profiles reached a plateau at a molar ratio of 0.5:1 (AChE:PrP) (Figure 4e). A subsequent addition of AChE did not further increase the average molecular weight of each fibrillar sample.

### Hup8TH reduces AChE-binding to PrP fibrils

To examine whether AChE binding to PrP fibrils depends, as for monomeric PrP, on the same AChE hotspot that can be targeted by Hup8TH, we studied by SLS the effect of Hup8TH on AChE interaction with amyloid fibrils obtained from full length, Δ23-99 and Δ100-120 PrPs. To this aim, we pre-incubated AChE with its inhibitor at a molar ratio of 1:20 (AChE: Hup8TH) at room temperature for 30 min. Then, we added the mixture to each of the three PrP fibrillar forms. Hup8TH strongly inhibited AChE binding to all PrP fibrils (Figure 4f, traces with asterisks). Moreover, AChE binding reduction was dependent on the PrP fibrillar form used and thus on the presence/absence of the two N-terminal sub-sites that are essential for this heterologous interaction. The resulting fraction of protein complexes estimated for full-length PrP fibrils in the presence of Hup8TH (~30%) corresponded approximately to the mean of the values obtained for fibrils formed from the truncated PrP variants.

### The interaction of AChE with PrP fibrils inhibits AChE enzymatic activity and enhances PrP fibril cytotoxicity

**Figure 5:**
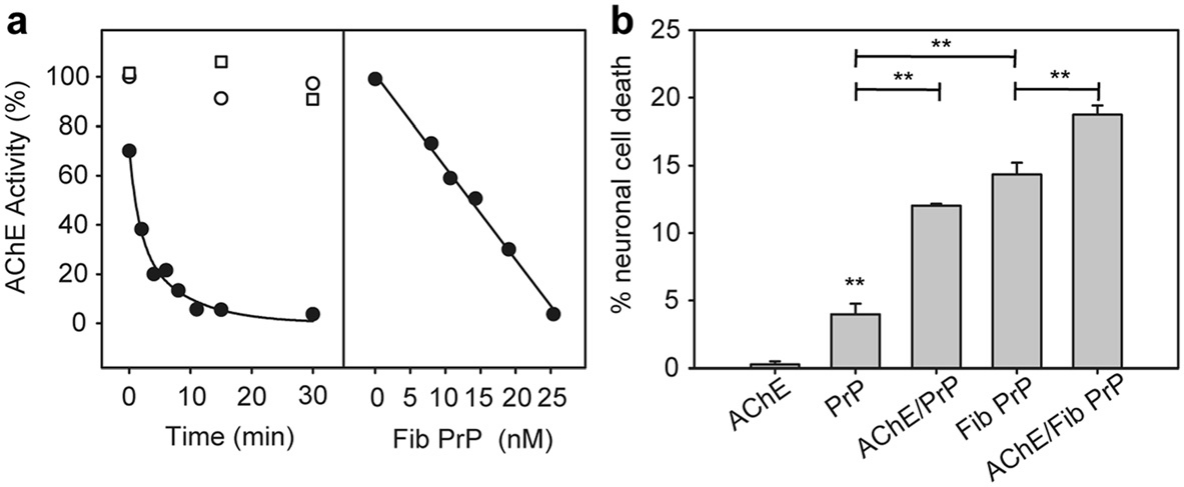
**The interaction of AChE with PrP fibrils inhibits AChE enzymatic activity and enhances the cytotoxicity of both monomeric alpha-helix-rich and amyloid fibril PrP conformers in primary neurons. a** PrP fibrils inhibit AChE enzymatic activity. Left panel: 7.3 nM AChE was pre-incubated for different periods of time without (open circles) or with 25 μm of monomeric (open squares) or fibrillar PrP (black circles) before initiation of the reaction with ACh. Right panel: Decrease in the observed AChE activity as a function of PrP fibril concentration. **b** Neurons were cultured for 2 days and then incubated with monomeric (PrP) or fibrillar (Fib) PrP isoforms (final concentration, 1 μM), 0.5 μM AChE or the PrP-AChE mix. Neuronal cell death was quantified and expressed as the percentage relative to the value of control cells (vehicle). Each set of data is the mean value ± S.D. (in percentage) of four experiments; five independent microscopic fields were counted for each experiment. **P < 0.005, one-way ANOVA. The Tukey test was used for post-hoc comparison after ANOVA.

We next determined whether PrP fibrils have any effect on the enzyme activity by assessing AChE enzymatic activity in the absence or presence of 25 μM monomeric or fibrillar PrP (3500 molar excess) (Figure 5a, left panel). Whereas no change was observed using monomeric PrP, AChE was completely inhibited after 30 min of incubation in the presence of fibrils. This inhibition was linearly proportional to the concentration of PrP fibrils (Figure 5a, right panel).

Finally, to investigate whether AChE interaction with PrP increases PrP toxicity we compared the cytotoxicity of soluble monomeric PrP, AChE and of the mix obtained after 30 min incubation, as well as of PrP amyloid fibrils and preformed amyloid fibrils associated with AChE by Hoechst nuclear staining of primary neuronal cells (Figure 5b). Monomeric PrP caused only a minor toxic effect, whereas the complex with AChE was three times more toxic, with a neuronal cell death rate comparable to that of PrP fibrils. The fibrillar morphotype arising from AChE association with PrP fibrils showed the highest cytotoxicity.

### Prion-infected AChE^+/−^ mice display slightly but significantly increased survival time

We then examined whether partial AChE gene knockdown in transgenic mice, which reportedly leads to a 25-35% decrease in AChE activity [52], could affect prion disease tempo *in vivo*. Following intracerebral inoculation of 139A prions, the mean survival time of AChE^+/−^ mice (160 ± 1 days, n = 9) was slightly but significantly longer than that of wild type mice (151 ± 3, n = 7)(p = 0.03 Mann–Whitney U test). However, at the terminal stage of disease, there was no difference in the brain accumulation and regional distribution of PrP^**Sc**^ in AChE^+/−^ and wild type mice.

### Prion infection selectively alters AChE levels *in vivo* and in cell cultures

**Figure 6:**
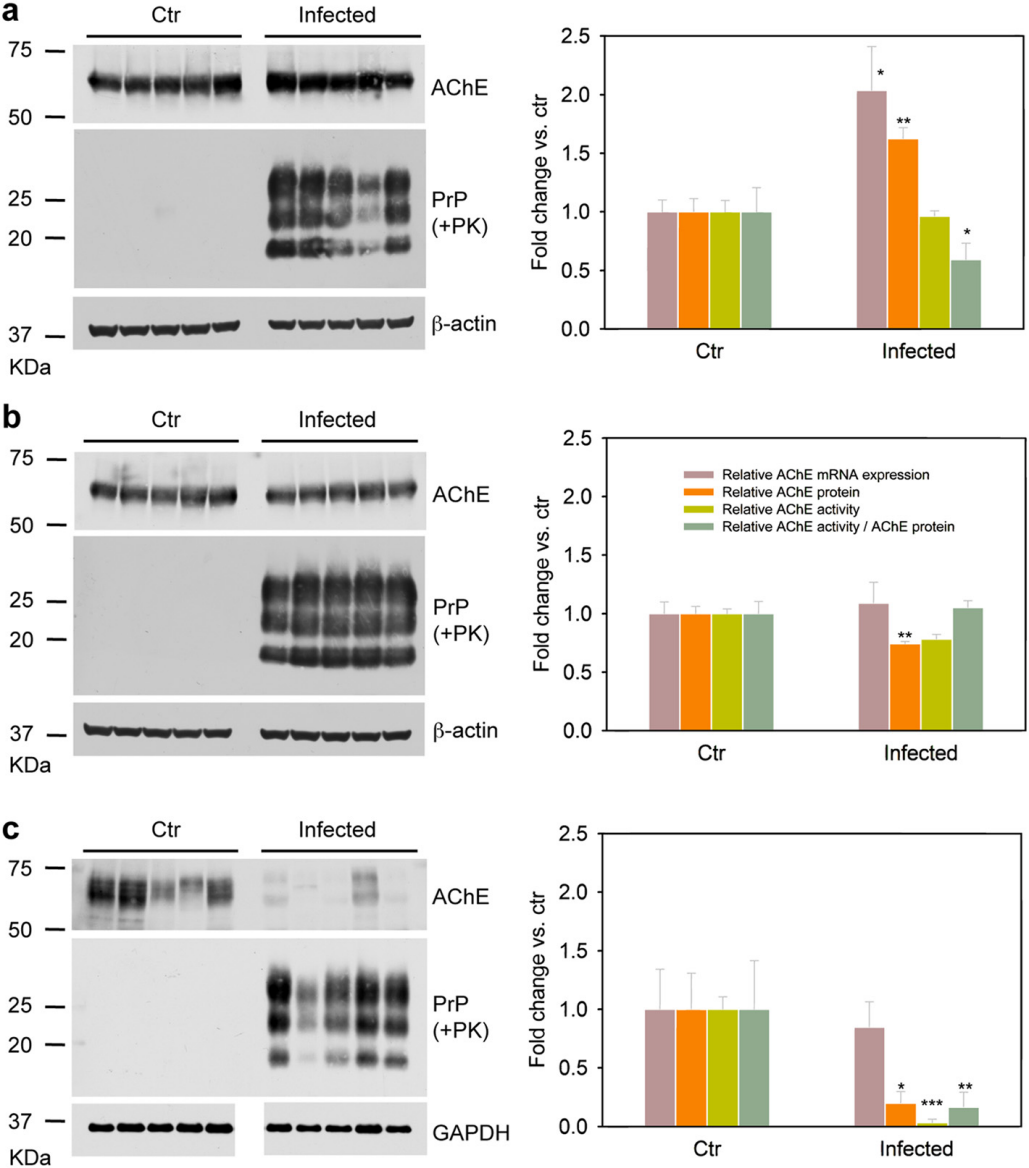
**AChE levels are altered in the brain and spleen of prion-infected mice.** *Left panels*: Western blot analysis of cerebral cortex-enriched homogenates **(a)**, brain extracts containing the remaining structures **(b)**, and spleen homogenates **(c)** prepared from *tg338* mice expressing ovine PrP (VRQ/VRQ PrP genotype) and infected with LA21K *fast* prions or not (controls, Ctr). All inoculated mice (n = 5) developed prion disease, as indicated by the presence of PrP^Sc^, and showed altered AChE protein levels compared to age-matched, mock-infected controls (n = 5). β-actin or GAPDH levels are shown as loading control. Molecular weighs (in kilodaltons) are indicated on the left of the blots. *Right panels*: Vertical bar charts comparing the levels of AChE transcripts, protein and enzymatic activity in control and infected mice. Values are presented as fold change relative to control mice. Data are the mean ± SEM obtained from the indicated number of mice. mRNA levels of total AChE transcripts were normalized to HPRT expression in the same cDNA preparations. Densitometric quantification of AChE protein was normalized to β-actin (brain) or GAPDH (spleen). *P < 0.05, **P < 0.005, ***P < 0.001 compared to controls (one-way ANOVA).

We tested whether AChE levels were altered *in vivo* in response to prion infection. We injected brain homogenates infected or not (mock) with LA21K *fast* prions in transgenic *tg338* mice that express ovine PrP (VRQ/VRQ PrP genotype). At the terminal stage of the disease, we measured the levels of AChE transcripts, protein and enzymatic activity in cerebral cortex-enriched homogenates (Figure 6a) and in the brain fractions containing the remaining structures (Figure 6b) from each infected mouse (n = 5) and age-matched mock-infected controls (n = 5).

RT-qPCR quantification showed that AChE mRNA levels were increased by two-fold in cortical-enriched extracts of infected mice compared to controls (Figure 6a, right panel). Conversely, the cholinergic ChAT mRNA levels were not significantly different (Additional file 4), suggesting that prion disease affects AChE in a specific manner. In addition, no difference in the splicing pattern of the *ACHE* gene was observed in the two groups. As expected, AChE-T mRNA (95%) was more abundant than AChE-R and AChE-H mRNAs (Additional file 4). *ACHE* gene up-regulation was accompanied by a comparable change in AChE protein expression in prion-infected brains compared to controls (Figure 6a). However, AChE activity was not increased compared to control mice, suggesting that prion infection causes AChE inhibition in these brain areas. Indeed, normalization of AChE activity values to AChE protein content, as estimated by western blotting, indicated that AChE catalytic activity was decreased by nearly 40% in the in cortical-enriched extracts of infected mice.

In the rest of the brain of infected mice, no increase in AChE mRNA levels was observed compared to controls (Figure 6b, right panel), and AChE protein and activity levels were reduced by 25% (Figure 6b). Therefore, the normalized AChE activity, calculated based on AChE protein content in these brain areas, was unchanged compared to control mice.

We then investigated whether the extra-cerebral AChE levels also were altered upon prion infection. We focused on the spleen, a secondary lymphoid organ in which prions can replicate and accumulate [53,54]. Despite noticeable inter-individual variability in the spleens of control mice, AChE protein level and enzymatic activity were very significantly lower (70% and 96% decrease, respectively) in spleen from infected mice compared to control (Figure 6c). Conversely, AChE mRNA levels were not changed compared to uninfected mice, indicating that AChE depletion during prion disease occurs via a post-transcriptional process and may be inherently linked to prion replication.

**Figure 7:**
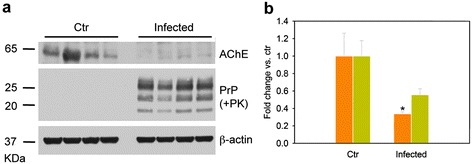
**Decreased AChE levels in prion-infected MovS6 cells. a** Western blot analysis of AChE levels in infected cells harboring PK-resistant PrP^S^. PrP^Sc^ accumulation was determined with the anti-PrP antibody Sha31b; the anti-β-actin antibody was used to control protein loading. Ctr, mock-infected cells. Molecular weights (in kilodaltons) are indicated on the left of the blots. **b** Quantification of AChE protein (orange bars) and enzymatic activity (green bars) in mock-infected (Ctr) and prion-infected MovS6 cells. Values are presented as fold change relative to control cells. Data are the mean ± SEM of four independent experiments for each condition. *P < 0.05 one-way ANOVA.

To investigate whether this phenomenon occurs *in vitro* in cultured cells, we examined AChE protein level and enzymatic activity in the neuroglial mouse cell line MOVS6 that is persistently infected with the 127S scrapie strain, which phenotypically resembles LA21K *fast* prions [45]. Consistent with our findings in the spleen, both AChE protein and activity levels were reduced in infected cells, by 66% and 45%, respectively, compared to control mock-infected cells (Figure 7).

## Discussion

Here, we provide conclusive evidence that AChE can be functionally linked to PrP misfolding. This novel function of AChE, which is present in the biological environment where PrP replication may occur naturally, brings crucial knowledge on prion structural changes and their potential pathobiological implications. In this work, we addressed the relationships between the formation of AChE/PrP hetero-assemblies and i) their cytotoxicity in primary neuronal cultures, as well as the extended survival of prion-infected AChE^+/−^ mice; ii) the interference of dual-site binding AChE inhibitors with prion accumulation; and iii) the altered AChE homeostasis in both nervous and lymphoreticular systems of prion-infected mice and in chronically infected cell cultures.

### AChE confers new properties to PrP amyloid fibrils

We show that AChE facilitates PrP aggregation and/or confers new structural properties to PrP amyloid fibrils. AChE incorporation into PrP amyloid fibrils substantially increases the PK-resistant core and specifically leads to a stronger ~16-kDa PK-resistant band, similar to that of naturally occurring PrP^Sc^ and to infectious synthetic prions [55,56]. This supports the idea that AChE facilitates a conformational rearrangement in which the PrP region between residues ~90 and 140 adopts a PK-resistant conformation, which is thought to be critical for PrP conversion to a PrP^Sc^-like molecule [43]. In addition, AChE assembly with PrP amyloid fibrils leads to enhanced hydrophobic exposure and significantly higher cytotoxicity in primary cultured neurons, compared to non-complexed PrP fibrils. Elevated neurotoxicity is also observed when AChE aggregates with the soluble PrP conformer. Thus, the longer survival observed in animals with lower AChE activity can be explained by a reduced amount of cytotoxic PrP morphotypes. The strongly reduced life expectancy and other abnormalities of AChE^−/−^ mice [57] currently do not allow studying the consequences of complete depletion of AChE on prion pathogenesis. It remains to be investigated whether AChE is present in scrapie-associated fibrils. Nevertheless, it is unlikely that under harsh biochemical conditions, such as detergent extraction and proteinase K digestion used to extract scrapie-assocaited fibrils [58], PrP will co-purify with potential binding partners that it complexes with inside the cell.

### Ionic interactions and a druggable aromatic-rich hotspot contribute to the AChE-PrP complex formation

The AChE-PrP interaction might be explained by the fact that AChE (i.e., each catalytic subunit constituting the enzyme) displays a very large dipole moment that is oriented approximately along the axis of the active-site gorge (Additional file 5a). This particular arrangement, which has an excess of negative charge on the surface near the entrance of the AChE catalytic gorge, renders the protein particularly prone to experiencing promiscuous electrostatic interactions. Therefore, the presence of an extended disordered N-terminal region with two positively charged sub-sites (Additional file 5b) in both the alpha-helix rich PrP monomer and amyloid PrP fibrils, might favor interaction with AChE. This heterologous interaction may partially mask the charge-charge repulsion between neighboring homo-molecules, thereby considerably increasing the local concentration of the protein, and favoring the rapid assembly. The lack of interaction with the N-terminal truncated PrP Δ23-120 variant further reinforces our hypothesis.

We also explain why dual-site binding AChE inhibitors are endowed with anti-prion activity. Our *in vitro* studies show that the specific tight interaction of Hup8TH (Additional file 5c), a dual-site binding AChEi, impairs AChE-PrP complex formation. Hup8TH, which is sandwiched between the PAS aromatic residues Trp286 and Tyr72 (in yellow in Additional file 5a) and the active-site gorge residues Tyr337 and Trp86 [59], occludes the AChE-PrP interface. These findings suggest that the precise assembly of the two oppositely charged proteins is critically dependent on such an aromatic-rich AChE hotspot. The proposed AChE-PrP binding mechanism is further supported by our results obtained using PrP fibrils lacking the 23–99 or 100–120 N-terminal PrP segments. The more positive charge distribution and rich aromatic character of the 23–99 region, compared to the 100–120 region, explain the different inhibition values observed with Hup8TH on AChE association to those fibrils. The anti-prion activity of Hup8TH in cell culture can therefore be ascribed to the displacement of the specific AChE-PrP interaction, further supporting AChE role in the formation of pathologically relevant PrP morphotypes.

### A Model of AChE-binding to PrP Amyloid Fibrils

Although we do not yet have the atomic structures of full length PrP amyloid fibrils, several biochemical and biophysical studies suggest that the PrP fibril spine, which goes approximately from residue 160 to 225, stacks in*-*register with parallel β-sheet strands spaced 4.8 Å along the fibril axis [60-64]. The rest of the protein (i.e., the non-structured N-terminus and the first α-helix) must somehow accommodate at the periphery of the spine.

AChE crystal structure [65] and our estimation of its molecular weight in solution (Additional file 6) suggest the formation of a dimer with the identified interaction hotspot in each catalytic subunit facing the opposite sides of the complex (Additional file 5a).

**Figure 8:**
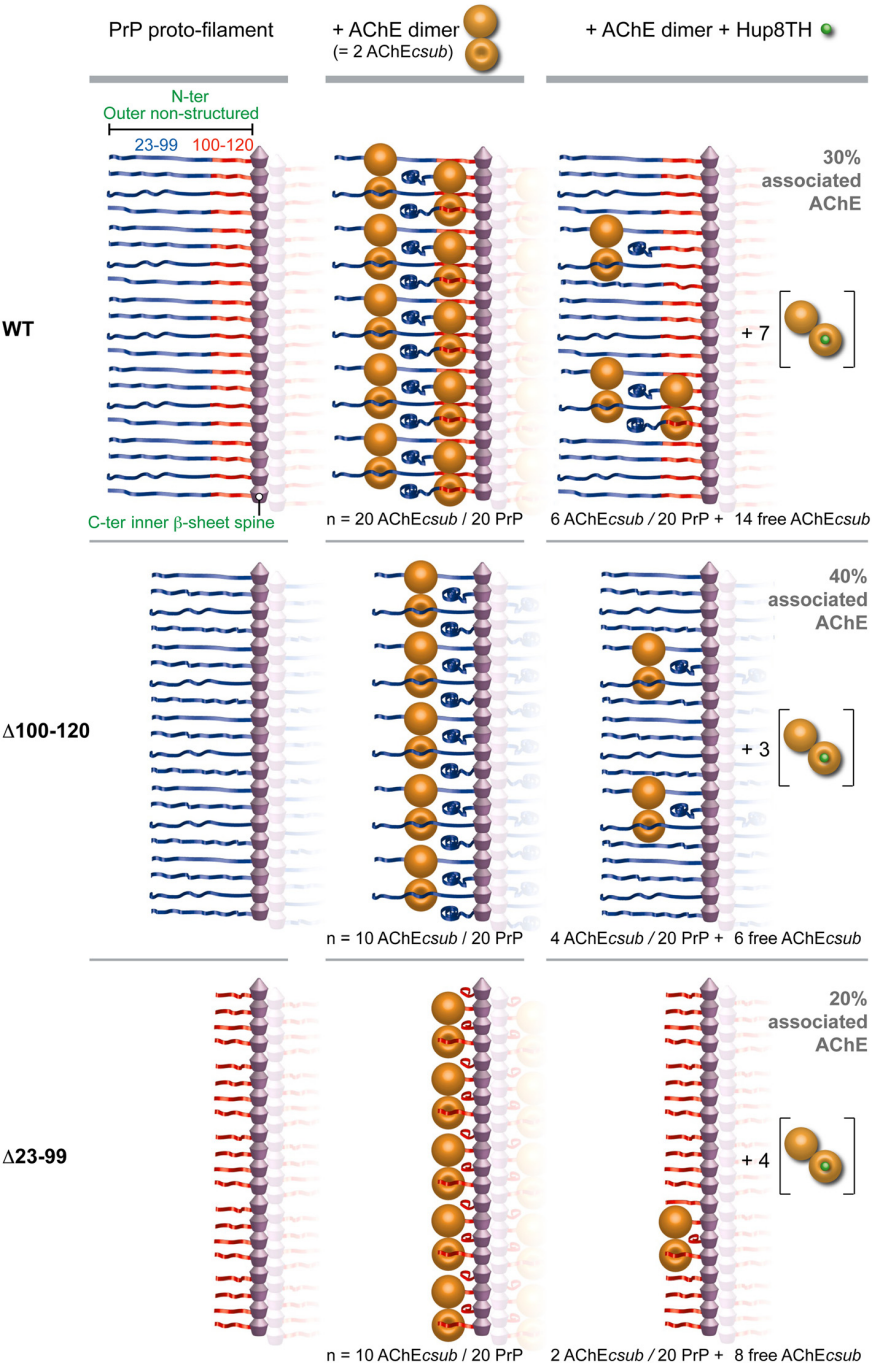
**Schematic representation of AChE binding to PrP amyloid fibrils.** This model of AChE interaction within PrP fibrils was built using full-length PrP and its deletion variants, and is based on the data on Hup8TH inhibition of aggregation. We considered that each PrP proto-filament consisted of an inner spine and an outer non-structured region that includes approximately the N-terminus and the first α-helix of PrP. Due to the specific structural features of the dimeric AChE molecule and the supposed regular structure of the PrP fibril, each AChE catalytic subunit (AChE*csub*) in the dimer may only interact with a non-contiguous PrP protomer in the protofilament. Thus, in the case of full-length PrP fibrils, AChE*csub* may associate with adjacent PrP protomers by alternately interacting with the N-terminal sites within residues 23 and 99 (in blue) and residues 100 and 120 (in red). As AChE binding to both sites in the same PrP protomer appears to be mutually exclusive, an equimolar interaction between proteins is therefore obtained. Conversely, for fibrils formed from truncated N-terminal PrP variants (∆100-120 and ∆23-99), which are characterized by the loss of one of the AChE binding sites, only alternate PrP protomers may be complexed with AChE. N-terminal steric hindrance between adjacent PrP protomers thus leads to a 0.5:1 stoichiometry. The inhibition data using Hup8TH are in agreement with the proposed model and reveal that the two PrP N-terminal sites have similar binding mechanisms, but different avidities. The number (n) of AChE*csub* (associated and free) and PrP protomers is also shown. Further details are specified in the Discussion section.

On the basis of these previous structural studies and the present results, AChE binding to PrP fibrils can be schematized as follows (Figure 8). In fibrils, AChE binds to the 23–99 and 100–120 N-terminal PrP segments that should be solvent-exposed and accessible to extrinsic biological factors. However, AChE binding to these two regions of a monomeric PrP or fibrillar PrP protomer appears to be mutually exclusive and, thus, only one of the two sub-sites in the PrP N-terminus can be occupied by AChE without any steric hindrance. The 0.5:1 (AChE:PrP) stoichiometry found for fibrils composed of truncated PrP variants can be attributed to the particular structure of the dimeric AChE molecule, together with the precise arrangement of contiguous PrP molecules in a protofilament. These structural constraints could make adjacent N-terminal PrP regions sterically unavailable for interaction with AChE. Accordingly, only every second PrP protomer can be complexed with an AChE catalytic subunit. In the case of full-length fibrils, AChE might circumvent the steric overlap to interact with contiguous PrP molecules by alternately interacting with the two N-terminal PrP sites. This view is reinforced by the fact that half the number of AChE catalytic subunits are needed to completely saturate the binding sites of amyloid fibrils made from the two truncated PrP variants compared to those made from the full-length form. In addition, the intermediate association value of AChE with full-length fibrils in the presence of Hup8TH (30%), compared to the values obtained for the truncated fibrils (40 and 20%), is in agreement with the different AChE avidity for each PrP site and further supports the proposed mechanistic association model.

### Prion replication affects AChE homeostasis

We found that AChE protein levels and *ACHE* transcripts are markedly increased in cerebral cortex-enriched homogenates from prion-infected *tg338* mice compared to mock-infected controls. Since, in this mouse model, both PrP^C^ distribution in the brain and PrP expression ratios between brain and spleen are very similar to those found in conventional mice [66], our results indicate that AChE homeostasis is intrinsically altered upon prion replication and may be related to compensatory mechanisms leading to *ACHE* gene expression up-regulation to counteract the decrease in AChE catalytic activity. These observations, together with the large decrease of both AChE protein levels and enzymatic activity in the spleen, where restorative mechanisms might be absent, indicate that AChE is not a mere bystander but might actively participate in prion disease pathogenesis. Furthermore, the finding that AChE homeostasis is altered also in the spleen of infected mice and in infected cell cultures, where PrP^Sc^ accumulation appears rather benign [67], dismisses the possibility that the changes observed are the results of neurodegeneration or an artefact due to intracerebral inoculation, and supports the hypothesis that they arise in response to PrP^Sc^ accumulation. Indeed, the presence of AChE in a variety of lymphoid and reticular cell types [68] and particularly in follicular dendritic cells of the germinal centers [69], the splenic cells in which prions might actively amplify, makes plausible a causative AChE-PrP interaction in prion pathogenesis. Nevertheless, the definitive functional link of AChE to this mechanism still remains to be determined. While the role of AChE in peripheral prion pathogenesis deserves further research, our findings are consistent with previous reports on reduced AChE activity in CSF samples of CJD patients and AChE altered glycosylation pattern in CJD CSF and brain tissue samples [32,33].

The specific mode of AChE interaction with PrP fibrils, as detailed before, may physically obstruct acetylcholine entry to the acylation site, and/or reduce its affinity by charge repulsion, thus explaining the inhibition of AChE enzymatic activity observed in the presence of fibrillar conformers, or in different prion-infected mice tissues. As mentioned above, the reduced AChE activity found in cortical-enriched extracts could stimulate AChE up-regulation in these brain areas. Alternatively, in subcortical brain regions and spleen, as well as in cell cultures, AChE complexed with PrP could become more susceptible or exposed to proteolytic degradation. The lack of compensatory mechanisms will lead to a reduction of the AChE load. Our model is also congruent with the cell- and tissue-dependent differences in N-terminal trimming of PrP^Sc^ [70]. Indeed, in both MovS cells and spleen tissue, PrP^Sc^ accumulates predominantly as trimmed species, but still contains the 100–120 N-terminal PrP sub-site to which AChE can bind.

## Conclusions

In summary, we report that AChE acts as an auxiliary molecule in PrP misfolding, leading to PrP morphotypes with potential relevance in prion diseases. Our finding that targeting a druggable site in AChE blocks the formation of these complexes is conceptually attractive and might be used for therapeutic strategies to target prion diseases and other protein misfolding disorders. Indeed, there is strong evidence for AChE involvement in Aβ-induced neuronal dysfunction and Aβ fibril formation [18,19,21,22,20]. In parallel, PrP also mediates Aβ neurotoxicity [71]. The functional involvement of both AChE and PrP in AD, together with the AChE-PrP assemblies described here, reveals intriguing similarities between AD and prion diseases that could bring fundamental insights into their origins and progression. In this context, AChE could act as a common denominator in the pathogenesis of both diseases and its pharmacological targeting could thus provide broader neuroprotection.

## Additional files

Additional file 1:
**Hup8TH does not interact with PrP.** Thermal unfolding of PrP in the absence (solid line) or presence (dotted line) of Hup8TH, at equimolar concentrations, recorded by DSC. Additional file 2:
**The macrostructure of the new AChE-induced PrP fibril morphotype is different from that of classical PrP fibrils.** Negative-stained transmission electron micrographs of pre-formed PrP fibrils alone (left panel) or mixed with AChE (right panel). The molar ratio used was 0.5:1 (AChE:PrP). Scale bar = 100 nm. Additional file 3:
**Altered digestion profile of the PK-resistant core of PrP fibrils after AChE binding.** a Silver staining following electrophoretic separation of PrP fibrils (10.5 μM) incubated or not with AChE (0.5 μM), before and after annealing. All fractions were PK treated. b Densitometric analysis of the PK-resistant core of the corresponding samples. Data are mean ± SEM values from four independent experiments. ***P* < 0.05, ***P* < 0.005, vs. Fib PrP alone (one-way ANOVA). ns = non significant. Additional file 4:
**Cholinergic ChAT mRNA levels and the splicing pattern of the** ***ACHE*** **gene are not significantly different in prion-infected mice compared to non-infected controls.** a Vertical bar chart comparing the levels of ChAT transcripts in cerebral cortex-enriched homogenates from control and infected mice. Values are presented as fold change relative to control mice. Data are the mean ± SEM for 5 mice/group. mRNA levels of total ChAT transcripts were normalized to HPRT transcript expression the same cDNA preparations. b Vertical bar chart comparing the levels of AChE T, H and R transcripts, relative to total AChE transcripts, in cerebral cortex-enriched homogenates from control and infected mice. Additional file 5:
**Highly asymmetric surface charge distribution in AChE and PrP.** Schematic representation of the three-dimensional structure and surface charges (red: negatively charged; blue: positively charged) of two different views of AChE (a) and PrP (b). The Coulomb potential was calculated by using the Poisson–Boltzmann equation as implemented in the Swiss-Pdb Viewer software and the crystallographic structures (1TQB for PrP, with a reconstituted N-terminal disordered segment; and 3LII for the dimeric recombinant human AChE). Each dimer consists of two AChE catalytic subunits (in light grey and in grey). The residues involved in the interaction with Hup8TH are in yellow. An excess of negative charge on the surface near the entrance of the AChE catalytic gorge and the positive character of the two N-terminal PrP segments (the 23–99 aa region is in blue and the 100–120 aa region in red) are shown. The figure was produced using Swiss-Pdb-Viewer. (c) Structure of the dual-binding site of the AChEi Hup8TH. Additional file 6:
**AChE molecular weight estimation by multi-wavelength light scattering coupled to size-exclusion chromatography.** AChE was loaded in a 60 × 0.78 cm TSK4000SW column using an AKTA purifier100 FPLC device. The column exit was connected to a homemade multi-wavelength light scattering device with a scattering angle of 112°. The Rayleigh number leading to the estimation of the molecular weight was determined by using carbonic anhydrase as standard. The estimated molecular weight corresponds to the mean value obtained from three different wavelengths: 406 nm (violet), 450 nm (blue) and 532 nm (red).

## Abbreviations

PrP: Prion protein
ACHE: Acetylcholinesterase
ACHEi: Acetylcholinesterase inhibitor
PrP^C^: Host cellular PrP conformer
PrP^Sc^: Misfolded pathological PrP conformer
AD: Alzheimer’s disease
Aβ: Amyloid β
PAS: Peripheral anionic site
CSF: Cerebrospinal fluid
CHO cells: Chinese hamster ovary cells
PK: Proteinase K
ThT: Thioflavin T
ANS: 8-anilino-1-naphthalene sulfonate
BSA: Bovine serum albumin
SLS: Static light scattering
DSC: Differential Scanning Calorimetry
TEM: Transmission Electron Microscopy
AChE*csub*: AChE catalytic subunit
