# Supplementary figures and images for: Interaction of prion protein with acetylcholinesterase: potential pathobiological implications in prion diseases
Source: Acta Neuropathol Commun. 2015 Apr 3;3:18. doi: 10.1186/s40478-015-0188-0 (PMC4383067; doi:10.1186/s40478-015-0188-0)

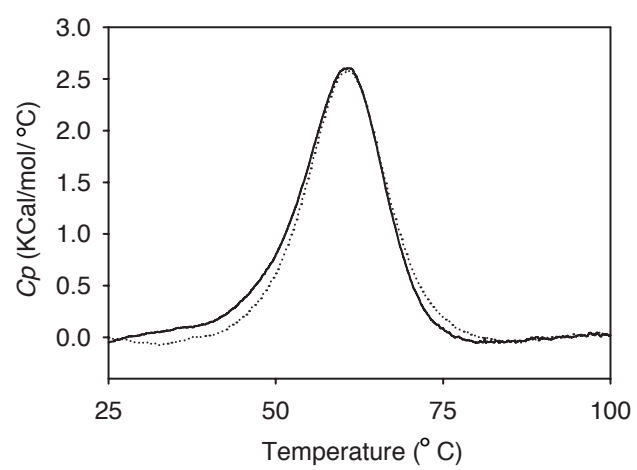

Supplement: Additional file 1: — Hup8TH does not interact with PrP. Thermal unfolding of PrP in the absence (solid line) or presence (dotted line) of Hup8TH, at equimolar concentrations, recorded by DSC. [file 40478_2015_188_MOESM1_ESM.pdf]

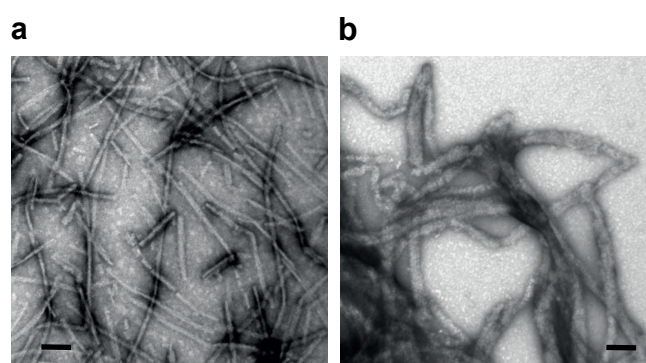

Supplement: Additional file 2: — The macrostructure of the new AChE-induced PrP fibril morphotype is different from that of classical PrP fibrils. Negative-stained transmission electron micrographs of pre-formed PrP fibrils alone (left panel) or mixed with AChE (right panel). The molar ratio used was 0.5:1 (AChE:PrP). Scale bar = 100 nm. [file 40478_2015_188_MOESM2_ESM.pdf]

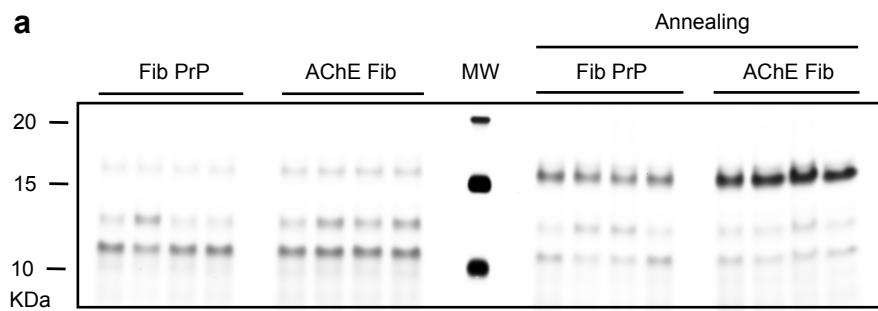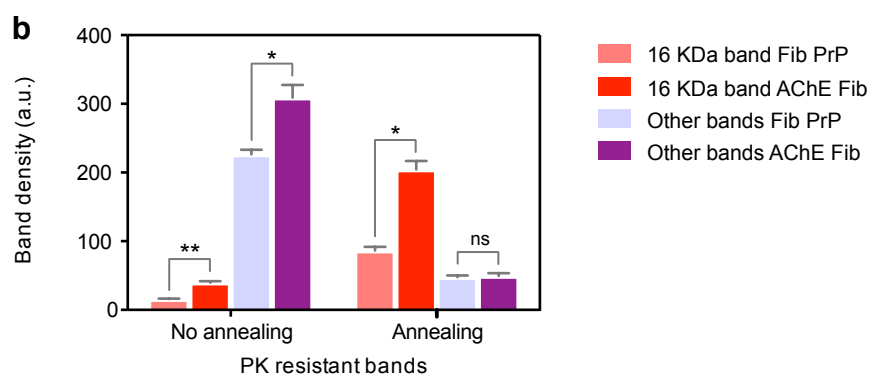

Supplement: Additional file 3: — Altered digestion profile of the PK-resistant core of PrP fibrils after AChE binding. a Silver staining following electrophoretic separation of PrP fibrils (10.5 μM) incubated or not with AChE (0.5 μM), before and after annealing. All fractions were PK treated. b Densitometric analysis of the PK-resistant core of the corresponding samples. Data are mean ± SEM values from four independent experiments. **P < 0.05, **P < 0.005, vs. Fib PrP alone (one-way ANOVA). ns = non significant. [file 40478_2015_188_MOESM3_ESM.pdf]

**a**

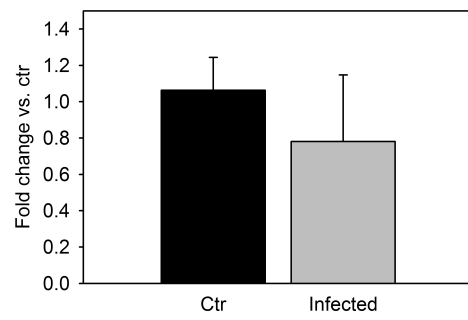

**b**

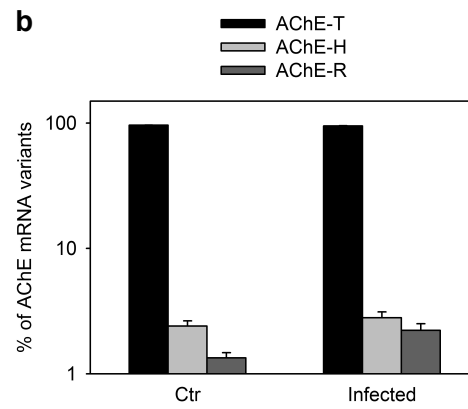

Supplement: Additional file 4: — Cholinergic ChAT mRNA levels and the splicing pattern of the ACHE gene are not significantly different in prion-infected mice compared to non-infected controls. a Vertical bar chart comparing the levels of ChAT transcripts in cerebral cortex-enriched homogenates from control and infected mice. Values are presented as fold change relative to control mice. Data are the mean ± SEM for 5 mice/group. mRNA levels of total ChAT transcripts were normalized to HPRT transcript expression the same cDNA preparations. b Vertical bar chart comparing the levels of AChE T, H and R transcripts, relative to total AChE transcripts, in cerebral cortex-enriched homogenates from control and infected mice. [file 40478_2015_188_MOESM4_ESM.pdf]

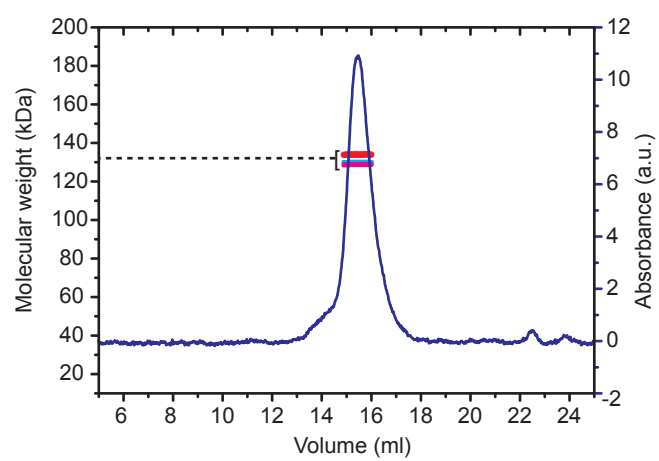

Supplement: Additional file 6: — AChE molecular weight estimation by multi-wavelength light scattering coupled to size-exclusion chromatography. AChE was loaded in a 60 × 0.78 cm TSK4000SW column using an AKTA purifier100 FPLC device. The column exit was connected to a homemade multi-wavelength light scattering device with a scattering angle of 112°. The Rayleigh number leading to the estimation of the molecular weight was determined by using carbonic anhydrase as standard. The estimated molecular weight corresponds to the mean value obtained from three different wavelengths: 406 nm (violet), 450 nm (blue) and 532 nm (red). [file 40478_2015_188_MOESM6_ESM.pdf]
