# Supplementary material for: Interaction of prion protein with acetylcholinesterase: potential pathobiological implications in prion diseases
Source: Acta Neuropathol Commun. 2015 Apr 3;3:18. doi: 10.1186/s40478-015-0188-0 (PMC4383067; doi:10.1186/s40478-015-0188-0)
Supplement: Additional file 5: — Highly asymmetric surface charge distribution in AChE and PrP. Schematic representation of the three-dimensional structure and surface charges (red: negatively charged; blue: positively charged) of two different views of AChE (a) and PrP (b). The Coulomb potential was calculated by using the Poisson–Boltzmann equation as implemented in the Swiss-Pdb Viewer software and the crystallographic structures (1TQB for PrP, with a reconstituted N-terminal disordered segment; and 3LII for the dimeric recombinant human AChE). Each dimer consists of two AChE catalytic subunits (in light grey and in grey). The residues involved in the interaction with Hup8TH are in yellow. An excess of negative charge on the surface near the entrance of the AChE catalytic gorge and the positive character of the two N-terminal PrP segments (the 23–99 aa region is in blue and the 100–120 aa region in red) are shown. The figure was produced using Swiss-Pdb-Viewer. (c) Structure of the dual-binding site of the AChEi Hup8TH. [file 40478_2015_188_MOESM5_ESM.pdf]

Figure 1 consists of two panels. The left panel shows a 3D surface representation of the 1918 H5N1 virus structure, with the HA1 domain highlighted in yellow. A yellow star indicates the location of the 222V mutation. The right panel shows a close-up of the HA1 domain, with the 222V mutation highlighted in yellow.
